# The Relation between Domain-Specific Physical Behaviour and Cardiorespiratory Fitness: A Cross-Sectional Compositional Data Analysis on the Physical Activity Health Paradox Using Accelerometer-Assessed Data

**DOI:** 10.3390/ijerph17217929

**Published:** 2020-10-29

**Authors:** Margo Ketels, Charlotte Lund Rasmussen, Mette Korshøj, Nidhi Gupta, Dirk De Bacquer, Andreas Holtermann, Els Clays

**Affiliations:** 1 Department of Public Health and Primary Care, Faculty of Medicine and Health Sciences, Ghent University, 9000 Ghent, Belgium; Dirk.DeBacquer@Ugent.be (D.D.B.); Els.Clays@Ugent.be (E.C.); 2 National Research Centre for the Working Environment, Lersø Parkallé 105, 2100 Copenhagen, Denmark; clr@nfa.dk (C.L.R.); ngu@nfa.dk (N.G.); aho@nfa.dk (A.H.); 3 Department of Occupational and Social Medicine, Holbæk Hospital, Part of Copenhagen University Hospital, Gl. Ringstedvej 4B, 4300 Holbæk, Denmark; melars@regionsjaelland.dk; 4 Department of Sports Science and Clinical Biomechanics, University of Southern Denmark, Campusvej 55, 5230 Odense, Denmark

**Keywords:** occupational health, compositional data, occupational physical activity, leisure time physical activity, physically demanding jobs, cardiorespiratory fitness, FEPA, CVD risk, aerobic workload

## Abstract

In contrast to leisure time physical activity (LTPA), occupational physical activity (OPA) does not have similar beneficial health effects. These differential health effects might be explained by dissimilar effects of LTPA and OPA on cardiorespiratory fitness (CRF). This study investigated cross-sectional associations between different physical behaviours during both work and leisure time and CRF by using a Compositional Data Analysis approach. Physical behaviours were assessed by two accelerometers among 309 workers with various manual jobs. During work time, more sedentary behaviour (SB) was associated with higher CRF when compared relatively to time spent on other work behaviours, while more SB during leisure time was associated with lower CRF when compared to other leisure time behaviours. Reallocating more time to moderate-to-vigorous physical activity (MVPA) from the other behaviours within leisure time was positively associated with CRF, which was not the case for MVPA during work. The results of our study are in line with the physical activity health paradox and we call for further study on the interaction between LTPA and OPA by implementing device-worn measures in a longitudinal design. Our results highlight the need for recommendations to take into account the different effects of OPA and LTPA on CRF.

## 1. Introduction

The health benefits of being physically active on a regular basis have been clearly established [1,2]. International guidelines therefore advise people to engage in at least 150 min of moderate- to vigorous-intensity physical activity (MVPA) per week to decrease the risk of cardiovascular disease (CVD) and all-cause mortality [3,4,5]. However, the beneficial effects of physical activity (PA) have mostly been demonstrated for PA performed outside the job setting, i.e., leisure time physical activity (LTPA), most notably in the context of sports and planned exercises [6]. PA that takes place in job settings, i.e., occupational physical activity (OPA), does not seem to have similar health benefits [7,8,9]. Additionally, it has been suggested that OPA might even be detrimental to cardiovascular health and a contributor to higher mortality [7,8,9,10]. The opposite health effects of LTPA and OPA are often referred to as the “PA health paradox” [11,12,13]. The PA health paradox might be accounted for by the different characteristics of LTPA and OPA, including the type, duration, intensity, and activity-rest-patterns of PA that differ between work and leisure time [11,14]. Furthermore, different physiological mechanisms might mediate between the opposite effect of the two types of PA on various health-related outcomes [15,16,17]. For example, the insufficient recovery time typical of OPA, together with the connected physiological damage, has been suggested as one potential mediating mechanism [14,18].

One potential underlying mechanism explaining the PA health paradox is that LTPA and OPA might have different effects on cardiorespiratory fitness (CRF). CRF (mlO_2_/min/kg) is defined as the ability of the cardiovascular and respiratory systems to supply oxygen to the muscles per kilogram of body weight [19]. Regular LTPA at a moderate- to vigorous-intensity combined with regular rest periods is known to maintain or even improve CRF levels [20]. By contrast, OPA and especially OPA which includes heavy lifting or prolonged standing, does not seem to have similar positive effects on CRF [21]. Studies have highlighted that OPA is often carried out at too low an intensity or for too long a duration, and with a too limited possibility of sufficient recovery breaks to be able to positively affect CRF levels [11]. Low levels of CRF are in turn strongly related to a spectrum of health-related problems, including CVD and all-cause mortality [22,23].

However, the international recommendations regarding PA, which can be summarized by the advice ‘move more and sit less’, do not differentiate between OPA and LTPA [4,5,6]. Workers already meeting the recommendations on PA through OPA might therefore mistakenly think that they already meet the recommendations on PA and think they can spend their leisure time in a sedentary fashion [24,25,26,27]. In reality, these types of workers might benefit from recommendations to take more sitting breaks during their work and to participate in leisure time MVPA to maintain or improve their CRF in order ‘to be fit for the job’ [28].

Yet, only a few studies have investigated the relation between OPA, LTPA, and CRF using technical measures of PA, and they often suffer from a number of methodological shortcomings and inconsistencies. First, previous studies have mainly relied on self-reported measurements to assess PA [21,29,30], resulting in low accuracy of the measurements and an increased likelihood of generating biased results. Second, findings have been notoriously inconsistent for workers involved in physically demanding jobs, who are likely to be impacted the most by the detrimental effects of OPA [31,32]. In particular, there is a lot of confusion regarding sedentary behaviour (SB), which is a major risk factor for cardiovascular morbidity and mortality [33,34,35]. On the one hand, sitting during work hours has been associated with increased levels of mortality [36], but results have also shown improved cardiovascular health when compared with other occupational physical activities, such as standing upright and/or walking [37,38]. Another limitation of the current literature is that the majority of studies have relied upon standard statistical regression techniques to investigate the associations between physical behaviours and CRF. This entails that activities such as sitting, standing, walking, or MVPA are conceptualized as individual entities, independent from one another. However, time-use of PA is by definition ‘compositional’ in nature, meaning that different physical activities are intrinsically co-dependent, as they inherently add up to ‘24 h a day’ or ‘100%’. In fact, it is impossible to increase the time spent on one particular activity without decreasing the time spent on another activity. Therefore, the use of Compositional Data Analysis (CoDA) is highly recommended [39,40], as it is based on the key assumption that the amount of time spent on one activity is only meaningful in light of the time spent on other activities. Only few studies in the field of OPA have hitherto adopted CoDA [41,42,43] and none for the study of the associations between OPA and LTPA on CRF. 

Thus, if we want to enable researchers to put forward better health recommendations for workers with high levels of OPA and increase our general understanding of the PA health paradox, it is of importance to further investigate the impact of domain-specific types of PA on CRF and the possible mediating factors. Furthermore, it is necessary to differentiate between various types of PA behaviours on the basis of criteria that pertain to the intensity, posture, and dynamicity of the behaviour. In particular, sedentary behaviour (SB), standing, low-intensity physical activity (LIPA), and MVPA should be differentiated as fundamentally different kinds of PA behaviours, regardless of whether they form part of OPA or LTPA. In fact, these kinds of PA are known to have different traits and also different effects on various health-related outcomes [22,37,44].

This study therefore aims to investigate the association between device-worn measures of physical behaviours during both work and leisure time and CRF among workers with high levels of OPA, while distinguishing between various types of PA and implementing a Compositional Data Analysis approach.

## 2. Materials and Methods 

The data used in this study were collected as part of the larger cross-sectional Flemish Employees’ Physical Activity (FEPA) study, which was conducted from February 2017 until June 2018. The participants in the sample of the FEPA study consisted of workers from seven companies within the service and production sector, i.e., a logistics and courier company, a food producing company, a hospital, and four manufacturing companies, all situated in Flanders, Belgium. Eligible participants for the FEPA study provided written consent to participate, were employed for at least 50%, were aged between 18 and 65 years, non-pregnant, and Dutch speaking. Further details about the study protocol, recruitment, and inclusion and exclusion criteria can be found in a recent protocol paper [45]. The FEPA study was approved by the Research Ethical Committee of Ghent University Hospital (number 2017/0129).

**Figure 1:**
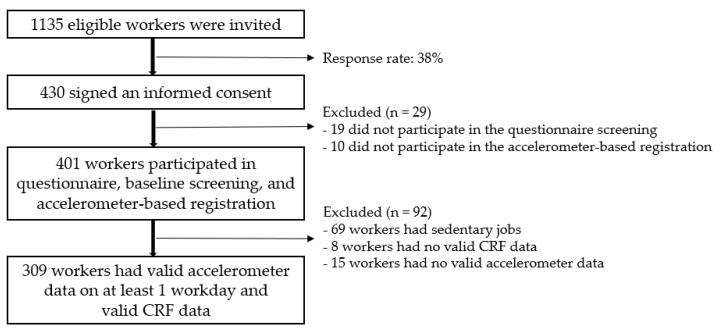
Flow chart of the recruitment of the study population.

This study focuses on a subsample of the FEPA study, including only those workers not having primarily desk-based jobs and thus excluding workers with sedentary jobs, e.g., administrative workers. Figure 1 provides a detailed overview of the recruitment process in our study. Eventually, a total sample of 309 workers was included in the further analysis. In addition to the level of OPA, there were only two other factors significantly different in the comparison of the workers with high OPA included in our sample and those with sedentary jobs (low OPA), i.e., the educational level and smoking behaviour. Workers with high OPA had a lower educational level and smoked on average less compared to the sedentary workers. For other factors, including age, sex, and body mass index (BMI), we found no significant differences between the two groups.

### 2.1. Measurements 

#### 2.1.1. Exposure Variables: OPA and LTPA 

OPA and LTPA were measured using data from two tri-axial Axivity AX3 (AX3 User Manual) accelerometers, one worn on the back and one on the right thigh. The accelerometers were worn for 2 to 5 consecutive working days, for 24 h a day. Simultaneously, workers were asked to keep a diary reporting on the beginning and end of their working day, their time of going to and getting out of bed, non-wear time, and the time of a reference measurement, i.e., standing still for 15 s in a neutral and upright position each day used to calibrate the two accelerometers. Further details about the measurements are provided in Ketels et al. [45].

The accelerometer data were downloaded using Axivity software (AX3-GUI, Omgui software) and analysed using a custom-made MATLAB software called Acti4 developed at the National Research Centre for the Working Environment, Copenhagen, Denmark, and the Federal Institute for Occupational Safety and Health, Berlin, Germany [46]. The Acti4 program is able to identify different types of PA and postures, including lying, walking, running, sitting, standing, moving, walking on stairs, rowing, and cycling, with high sensitivity and specificity [46]. To identify OPA and LTPA, the information retrieved from the diary was used to classify the accelerometer data into three time periods, i.e., leisure time, work time, and time spent in bed. The work period was defined as the hours spent in the primary occupation, i.e., during work, whereas leisure time, i.e., the time before and after work, was defined as time away from work, not including sleep. Non-wear periods were excluded according to previously defined criteria [45]. Only participants with measurements of both work and leisure time for at least one valid day were included for further analysis. A valid day was defined as including at least 4 h of work and leisure time, or 75% of the average reported work and leisure time.

SB was defined as time spent sitting and/or lying. Time spent standing was retrieved directly from the accelerometer data. Time spent moving (a standing position with small movements but without regular walking) and walking slowly (≤100 steps per minute) was combined to estimate time spent on LIPA. MVPA was defined as the time spent running, walking on stairs, and fast walking (>100 steps per minute). The mean time spent on three activities, i.e., SB, LIPA, and MVPA, was calculated for both work and leisure time in minutes per day. The accelerometers also detected other physical behaviours, such as cycling and rowing, but these behaviours hardly occurred during either work (0 ± 1 s/day for cycling and 1 ± 5 s/day for rowing) or leisure time (4 ± 10 s/day for cycling and 1 ± 3 s/day for rowing), and were therefore removed from the dataset for further analysis. 

#### 2.1.2. Outcome Variable: Cardiorespiratory Fitness (CRF)

To measure the level of CRF, the Harvard step test (HST) [47] was used. The Harvard step test is a single-stage test used to determine the physical fitness index (PFI) [48]. Participants were asked to step up and down on a bench (33 cm for women and 40 cm for men) for 5 min [49]. The rhythm was indicated by a metronome with a stepping rate of 22.5 steps/minute. Before the test, the participants received the necessary information about the testing procedure, and they had a one-minute practice session to become acquainted with the protocol. After the test, participants were asked to sit down, which was followed by a measurement of three recovery heart rates (in beats per minute) using a polar device (Polar A300 HR, Kempele, Finland) with a one-minute epoch. The three heart rates were used to calculate the physical fitness index (PFI), which is determined by the following equation: PFI% = (Duration of exercise in seconds × 100)/(2 × (recovery heart rate 1 + 2 + 3)) [48]. For example, if a total test time amounted to 300 s, and the three recovery heart beats were respectively 100, 90, and 80, the physical fitness score would amount to (300 × 100)/(2 × 270) = 55.55. The PFI was used as a continuous measure to estimate the CRF levels of the participants.

#### 2.1.3. Baseline Characteristics and Confounding Variables

Baseline characteristics were assessed by a self-administered questionnaire and baseline clinical measures. The questionnaire data included socio-demographic data, i.e., age, sex, marital status, education level, and occupational information, including seniority, work schedule, and working hours per week. Smoking status was classified as ‘non-smoker’ or ‘current smoker’ and educational achievement was categorised in three levels, until primary school was coded as ‘low’, secondary school and/or 1 to 2 years of specialisation as ‘medium’, and university or university college as ‘high’. The Job Content Questionnaire (JCQ) was used to assess specific measures of physical exertion, i.e., physical effort, heavy physical work, a lot of rapid work, difficult body positions, and difficult head and arms positions. The items on the JCQ had to be rated by means of a 4-point Likert scale ranging from ‘completely disagree’ to ‘completely agree’. The mean score of the 5 items was then calculated, which was used to capture the overall physical work demands of the participants. Height (m) and body weight (kg) were measured by using a SECA 704-column scale (SECA Medical Measuring Systems and Scales, Birmingham, UK; scales 701/704). Based on the outcome of height and weight, the corresponding body mass index (BMI, kg/m^2^) was calculated and classified following World Health Organisation (WHO) guidelines as ‘underweight’ (<18.5 kg/m^2^), ‘normal weight’ (18.5–24.9 kg/m^2^), ‘overweight’ (25–30 kg/m^2^), and ‘obese’ (>30 kg/m^2^). The use of any prescribed medication was determined by asking the participants the following question: “Have you over the last 2 weeks been taking medication for one of the following health conditions? (please tick the relevant box): heart disease, high blood pressure, high cholesterol, respiratory disease, mental illness, cancer, diabetes, and/or other”. 

### 2.2. Statistical Analyses

All analyses were conducted in R version 3.6.1 (RStudio, Vienna, Austria) [50], using the ‘Compositions’ [51] and ‘zCompositions’ [52] packages. The data used for this study were compositional in nature (cf. introduction) and provided relative information. Therefore, a CoDA approach was implemented [39,53]. Because the number of working hours is generally fixed and there is limited possibility to change behaviours between work and leisure time, both work and leisure time were treated as two separate 4-part compositions, consisting of time spent on SB (sitting and lying), standing, LIPA (moving and slow walking), and MVPA (fast walking, running, and walking on stairs).

#### 2.2.1. Compositional Descriptive Statistics

As a measure of central tendency, the geometric mean for time-use of OPA and LTPA was calculated and normalised or closed to 100% and 460 min, i.e., the mean accelerometer-assessed amount of minutes for both work and leisure time, to obtain the so-called ‘compositional mean’ [39].

#### 2.2.2. Log Ratio and Multiple Regression Analysis 

The 4-part OPA and LTPA compositions were transformed to three isometric logratios (ilrs) separately for OPA and LTPA composition. For calculation of ilrs, we used a sequential partition of one part to the remaining parts. This means that the first log ratio has the first compositional part as its numerator, and the geometric mean of all other compositional parts as its denominator [40]. We constructed four ilrs sets (3 ilrs in each set), corresponding to four different types of behaviours, i.e., SB, standing, LIPA, and MVPA, by rotating the sequence of the compositional parts so that each part was iteratively considered as the first compositional part. In this way, the relative importance of each part was sequentially represented in the first ilr-coordinate. Subsequently, four multiple linear models were constructed, one for each ilr-coordinate set, to estimate the association between each relative time spent on each behaviour during work and leisure time separately (exposure variables expressed as ilr-coordinates) and CRF (outcome variable). Confounders were included in our analysis based on insights expressed in previous literature and on a number of theoretical assumptions regarding their possible influence on work behaviours, leisure time behaviours, and CRF [54,55,56,57]. The models were adjusted for age, sex (reference group = female), educational level (reference group = low education), BMI, smoking (reference group = smoker), MVPA (MVPA work expressed as ilr for the relation of LTPA and CRF and MVPA leisure expressed as ilr for OPA and CRF), and self-reported heavy physically demanding tasks during work. Missing data were not imputed, which entails that participants with missing data for any of the variables used in the models were excluded from the further analysis. For the first ilr-coordinate (ilr₁) of each coordinate set, the beta coefficients (β), standard error of beta, t-statistic, and *p*-values were reported.

#### 2.2.3. Compositional Isotemporal Substitution Analysis

In order to interpret the beta coefficients (β) from the multiple linear regression, we used compositional isotemporal substitution models based on the methodology explained in Dumuid et al. [58] and Gupta et al. [42]. Overall, this method helped in determining the difference in CRF (i.e., physical fitness index) by reallocating time from one behaviour to the remaining behaviours, while keeping the total time at work and in leisure time constant. The substitution models were performed within each domain (work and leisure time), instead of performing inter-domain reallocations [42], given the unlikely transition of physical behaviours between work and leisure time. These isotemporal substitution models were executed in three steps. First, compositional mean of OPA and LTPA was determined. Second, new OPA and LTPA compositions were constructed based on the one-to-remaining reallocation method, reallocating time between the work behaviours and leisure time behaviours (10, 20, and 30 min). Third, the estimates obtained from the multiple regression analysis were used to determine differences in CRF (i.e., physical fitness index) based on reallocation between behaviours within the OPA and LTPA, respectively. The estimated differences for CRF were calculated for time reallocations of 10, 20, and 30 min and 95% confidence intervals (CI) were obtained (Supplementary Tables S1 and S2). When 95% CI did not include zero, the change was considered as significant. Additionally, in order to obtain more detailed information regarding which specific physical behaviour played a role, one-to-one reallocations were calculated in addition to the one-to-remaining reallocations. This entails that fixed durations of time, i.e., 10, 20, and 30 min, were reallocated from one specific behaviour to another one, while the time spent on the remaining types of behaviour was kept constant.

## 3. Results

### 3.1. Descriptive Statistics 

Descriptive statistics for the baseline characteristics in our sample of 309 workers are shown in Table 1. The mean age was 38.5 (±11.2) years, 57.6% of the participants were women, and more than half of the participants graduated from university and/or university colleges (51.1%). Most of the participants worked in the manufacturing sector (49.5%), 64.1% worked in shifts, and the average working time (self-reported) was 36.9 h per week. The participants had a mean BMI of 24.7 (±3.9) kg/m^2^, 22% smoked, and 5% used medication for any heart condition and/or high blood pressure. On average, workers provided 2.8 (±0.9) valid accelerometer-assessed working days, with an average wear period of 7 h and 47 min (±1 h 5 min) during work, 7 h 38 min (±1 h 38 min) during leisure time (sleeping time excluded), and slept an average of 7 h 27 min (±1 h 8 min).

The compositional means analysis (Table 2) revealed that most work time was spent standing (39.5%), followed by SB (29.3%). During leisure time, the workers spent the majority of their time in SB (61.5%), followed by standing (20.4%).

### 3.2. Main Analysis

The regression coefficient (Table 3) for ilr₁ indicated that more SB time during work time was associated with higher CRF levels when compared relatively to time spent standing, LIPA, and MVPA (β = 4.66; *p* < 0.01), whereas more SB during leisure time was associated with lower CRF levels when compared relatively to the other leisure behaviours (β = −11.84; *p* < 0.001). A significant positive association was observed between more MVPA during leisure time relative to the other leisure time behaviours and CRF levels (β = 12.99; *p* < 0.001).

**Figure 2:**
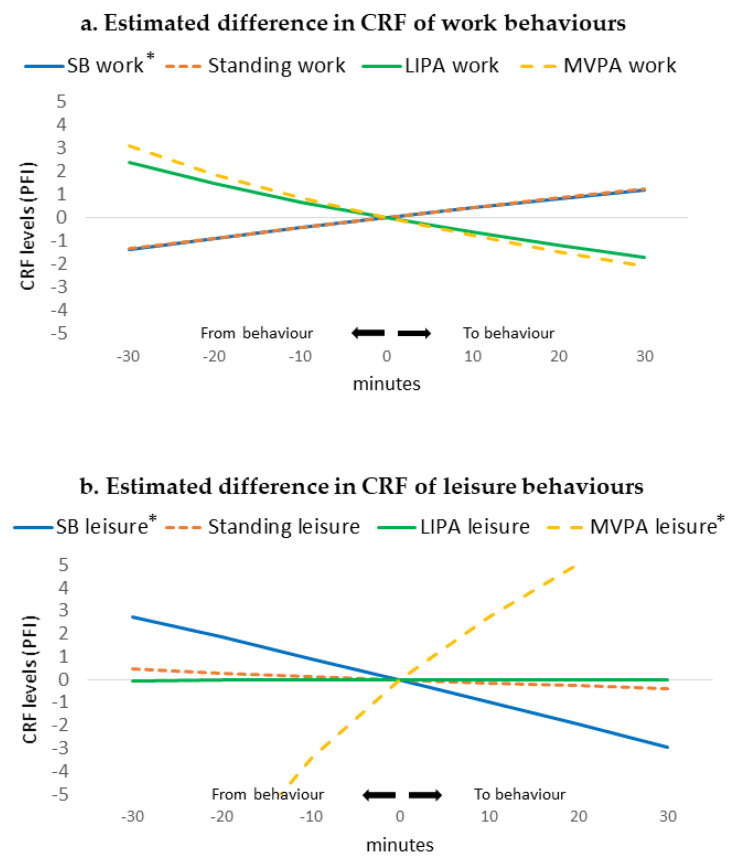
Estimated difference in CRF (based on the physical fitness index) levels associated with one-to-remaining reallocations of different behaviours during work (**a**) and leisure-time (**b**) among 309 workers. X-axis represents the amount of minutes reallocated from a behaviour to remaining behaviours within each domain, Y-axis represents the change in CRF levels. * significant at *p* < 0.05. CRF = cardiorespiratory fitness; PFI = physical fitness index; SB = sedentary behaviour; LIPA = low-intensity physical activity; MVPA = moderate-to-vigorous physical activity. The confidence intervals of the estimates are presented in Supplementary Table S1.

The results of the compositional isotemporal substitution analysis (Figure 2) demonstrated that more time spent in SB at work, relative to other work behaviours, was associated with higher CRF levels, while more time spent in SB during leisure time, relative to other leisure behaviours, was associated with lower CRF levels (Figure 2 and Supplementary Table S1). In particular, the one-to-one reallocations revealed that by replacing, for example, 20 min SB at work by MVPA, the physical fitness index decreased with 1.85 units (95% CI = −3.60, −0.11), whereas replacing 20 min SB during leisure time with MVPA was associated with a 5.22 (95% CI = 2.97, 7.47) higher physical fitness index (Supplementary Table S2). Figure 2 shows furthermore that more time spent in MVPA during work, relative to the other behaviours, was not significantly associated with lower CRF levels, while more MVPA during leisure time was significantly associated with higher CRF levels. One-to-one reallocations showed that reallocating 20 min from SB or standing at work to MVPA was associated with a 1.85 (95% CI = −3.60, −0.11) and 1.75 (95% CI = −3.36, −0.15) lower physical fitness index respectively, while reallocating 20 min of all the other behaviour separately (i.e., SB, standing, and LPA) to MVPA during leisure time was associated with higher CRF levels.

Reallocating time to standing, LIPA, and MVPA during work hours separately from the other work behaviours was not significantly associated with a change in CRF levels. Likewise, during leisure time, spending more time standing and LIPA, relative to all other leisure time physical behaviours, was not significantly associated with a change in CRF levels.

### 3.3. Results of the Sensitivity Analysis 

Due to the potential influence of medications taken for heart conditions and/or high blood pressure on the relation between OPA/LTPA and CRF [59], the analyses were repeated excluding the participants who used these kinds of medications. The new subsample included 293 participants and the results of the sensitivity analysis were in accordance with those obtained in the overall sample.

## 4. Discussion

The results of our study are, generally speaking, very much in line with the pattern known as the PA health paradox. Reallocating more time to SB from physically active behaviours during work time resulted in a significant association with higher CRF levels, whereas reallocating more time to SB from physically active behaviours during leisure time resulted in a strong negative association with CRF levels. Based on the one-to-one reallocations, reallocating 10 min from MVPA during work time to SB or standing separately was associated with an increase in CRF levels. This was in direct contrast with the finding that reallocating more time to MVPA from the other behaviours within leisure time resulted in a strong positive association with CRF levels.

### 4.1. The Physical Activity Health Paradox 

Our results confirm the well-known positive association between leisure time spent in MVPA and higher CRF levels [22,60]. The effect of MVPA on CRF exhibits a dose-response relationship, meaning the higher the amount or intensity of PA, the greater the increase in CRF levels [22,61]. In our study, this was manifested in the absence of a significant relationship between LIPA and CRF, and the presence of a significant relationship between MVPA and CRF. This finding is in line with studies which have suggested that it is especially the increase of the intensity of PA that results in more benefits for CRF, compared, for example, to the increase of the duration or frequency of PA bouts [62,63].

The results of our study revealed that more MVPA during work at the expense of the remaining work behaviours was not significantly associated with CRF levels, while the one-to-one reallocations revealed that reallocating 10 min from MVPA to SB or standing separately resulted in a small increase of CRF levels, which seems to entail that lowering the amount of MVPA during work is beneficial for the cardiorespiratory system. In contrast to the clear relationship between MVPA during leisure time and CRF levels, evidence regarding the association between MVPA at work and CRF remains inconsistent in the literature. A large cohort study among 4715 men and women showed that workers engaged in heavy physical work (self-reported) exhibit higher levels of CRF, as assessed with a step test, compared to young men engaged in lighter work [30]. Moreover, various other studies have demonstrated significant positive associations between OPA and VO_2_max [64,65]. 

Nonetheless, our findings are in full agreement with a strand of more recent studies which also reported different associations between domain-specific PA and CRF levels. The cross-sectional study of Bahls et al. [31] revealed a positive association between self-reported LTPA, sports, and CRF, whereas self-reported work-related PA was not shown to be beneficially associated with CRF [31]. Similarly, a study among manual and non-manual Swiss employees also revealed no independent association between technically assessed OPA at any intensity, using SenseWear mini armband, and VO_2_max, while intensive LTPA was positively correlated with VO_2_max levels [66]. In addition, the recent cross-sectional study of Zeiher et al. [32] showed that especially women engaged in physically demanding OPA who did not participate in LTPA had the highest likelihood of having low levels of VO_2_max. For men, the combination of low LTPA and low OPA showed the strongest negative relation to VO_2_max.

In sum, some studies have been unable to reveal an association between OPA and CRF, whereas other studies have found both positive and negative associations between OPA and CRF. We surmise that the reason for the huge discrepancy in the results might be related to large variation in levels of OPA among study populations and above all, to the different methodologies used to assess OPA. First, in the majority of studies, self-reported questionnaires were used to assess OPA, which are prone to recall and social desirability bias [67]. Furthermore, studies relying on technical measurements, i.e., accelerometers, mostly equate OPA with MVPA during working hours, i.e., the sum of running, walking, stair climbing, and to a lesser extent, cycling and rowing. MVPA of this form could positively affect CRF. However, accelerometers are not able to identify tasks that are particularly relevant, such as heavy lifting, working in awkward or static positions, and arms elevated above head. These specific tasks are perhaps more an expression of strength than of aerobic capacity and are captured better in studies that are based on self-reported questionnaires.

This study brought to light various interesting results regarding SB which mainly confirms that the relation between SB and CRF differs during work and leisure time, in line with the general pattern of the PA health paradox. Reallocating time to SB during work was positively associated with CRF levels. This stands to some degree in contrast to previous studies reporting that occupational sitting in office workers is associated with higher risks for fatal and non-fatal CVD [36]. This contrast can potentially be accounted for by the different exposure to OPA of our participants and the ones in the aforementioned study. For workers with physically demanding jobs, increased SB can provide a form of rest, whereas it might create an overload of SB for office workers. Our study therefore supports the idea of incorporating more rest breaks during the workday in physically demanding jobs for their potential beneficial health effects [28]. In sharp contrast, reallocating time to SB during leisure time resulted in negative associations with CRF levels. This is in line with other studies which showed that increasing levels of SB during leisure time is associated with lower levels of CRF [68], and thus a risk factor for cardiovascular and other chronic diseases [69,70].

Our results thus emphasize the need for more high-quality research on the PA health paradox in order to create a better scientific underpinning to differentiate between OPA and LTPA in future PA guidelines. It is furthermore highly relevant to take into account that our results relate to workers involved in physically demanding jobs. Guidelines for this group should be differentiated for workers with sedentary jobs. We would therefore recommend workers in physically demanding jobs to have more rest breaks during their work, which runs contrary to the general public health guidelines ‘to sit less and participate more in MVPA’ [28]. There is great need for clear guidelines that are tailored to the specific needs of various types of workers, because long durations of MVPA in combination with insufficient rest breaks might cause detrimental health effects for workers with physically demanding jobs [14].

### 4.2. Potential Underlying Mechanisms 

The positive association between MVPA during leisure time and CRF might be explained by the optimal type, duration, intensity, frequency, and rest periods of these types of PA. LTPA is mostly characterised by dynamic movements of large muscle groups over short time periods at high intensity levels (at least 60% of maximum oxygen consumption), which leads to an acute increase in heart rate, blood pressure, ventilation, and energy expenditure. These acute changes in combination with sufficient recovery will lead to long-term peripheral and metabolic changes, and thus lower 24 h heart rate and blood pressure levels, as well as higher CRF and decreased risk for cardiovascular problems and mortality [16,22,31,71,72]. 

In contrast, OPA is generally characterised by prolonged exposure to static or anaerobic PA during many hours a day with limited opportunities to take breaks. These long hours of PA at insufficient intensity will lead to prolonged elevation of heart rate and blood pressure, which is known to put tremendous strain on the cardiovascular system, including erosion of the endothelium and increased stress on the arterial wall, causing atherosclerosis [17] and/or sustained raised blood pressure [73]. Workers involved in physically demanding jobs also have limited control over their work speed and duration, leading to a lack of sufficient rest breaks or recovery, which may cause fatigue and exhaustion. The sustained fatigue in combination with insufficient intensity levels at work may result in a lack of CRF improvement [27], leading to increased risk for CVD and mortality [74].

### 4.3. Strengths and Limitations 

To the best of our knowledge, this is the first study that has implemented the statistical CoDA technique for investigating domain-specific PA behaviours in relation to CRF. Consequently, the use of the innovative CoDA statistical approach is a major strength of this study. This statistical method enabled us to take into account the compositional nature of the data. A second major methodological strength is the use of accelerometer-based measures to assess OPA and LTPA, which is essential for avoiding the self-reported bias associated with the use of questionnaires and provides valid measurements of different PA behaviours over multiple days, 24 h/day. The use of Acti4 software, which has shown to identify different postures and activities with high sensitivity and specificity, was a particular advantage in this study [46,75]. Finally, the size of our sample of workers involved in physically demanding jobs, including both men and women, is an important strength contributing to the external validity of our findings.

Our study also has limitations that need to be considered. Notwithstanding the important advantage of using two accelerometers, we were not capable to measure strenuous postures such as forward bending of the back and repetitive movements of the arms above the head, which are common during working hours. These specific tasks may be associated with lower levels of CRF, due to their detrimental impact on blood pressure and the cardiovascular system in general [42,73]. The use of even more accelerometers, preferably 4 [76], is required for a more comprehensive assessment of such specific physically demanding postures and tasks in order to further explore their relations with CRF. Also, the use of a cross-sectional design does not allow for inferring any type of causality, meaning that an inverse direction of the association between OPA/LTPA and CRF can in principle not be ruled out. While regular PA might increase CRF levels, it is also possible that participants who have genetically inherited a lower CRF may tend to be less active [77]. 

The use of convenience sampling may have limited the representativeness of our sample. However, variation in exposure to OPA and LTPA within the sample is the most crucial factor in detecting associations. Participation to the study was voluntary and may have led to selection bias. This issue is quite relevant since we cannot rule out that participants were younger and fitter than their non-participating colleagues. Also, the recruitment strategy through workplaces and the necessity of conducting all measurements during working hours may lead to selection bias at the company level, with only companies with higher resources choosing to participate. Only working days were measured, which means that the composition of leisure-time activities during non-working days might be different in comparison to working days.

Due to our relatively large sample size, CRF was estimated using a submaximal step test, namely the Harvard step test [48,49], and not by means of a maximal incremental cycle ergometer protocol. The maximal incremental cycle ergometer test is arguably the most valid method of assessing VO_2_max of workers, although it requires participants to be familiar with biking, but is rarely applied outside medical settings. Although the Harvard step test is easily applied and at lower cost, there may also be disadvantages of using this submaximal step test. It is known that workload increases with body weight. Consequently, the step test is well feasible for healthy persons in a good physical condition, whereas it might require near-maximal effort for less fit workers.

## 5. Conclusions

This study was the first to investigate the relation of OPA and LTPA with CRF by relying on accelerometer-based measures of physical behaviours. By focusing on CRF as the relevant health-related outcome and by implementing a CoDA perspective for the analysis of our data, the study filled several gaps in the current literature. Our results emphasize the need for taking the domain-specific nature of PA into account to understand its relation to CRF. MVPA was positively associated with CRF during leisure time only, which was not the case for MVPA during work time. In addition, more relative SB during work was positively associated with CRF among workers exposed to high OPA, which highlights the potential benefit of implementing more rest periods for workers exposed to high OPA. Hitherto, guidelines usually do not differentiate between OPA and LTPA in their recommendation to participate in at least 150 min of moderate PA per week, regardless of the OPA level. At least for workers exposed to high OPA, prevention could focus on finding a good balance between level of OPA and individual capacity (i.e., CRF), which can be achieved by either reducing the absolute physical workload, increasing CRF, increasing recovery time, or a combination of these factors.

Further research is called for to understand the specific characteristics of OPA and LTPA, and the underlying mechanisms involved in the opposite effects of LTPA and OPA on overall health, referred to as the PA health paradox. Future studies should focus on examining which specific combination of LTPA and OPA can lead to the most favourable health effects and which combination to the most detrimental, before recommending specific prevention measures. Implementing technical measures in a longitudinal design is highly called for as this can give us some insights in the causal relations involved. Finally, intervention studies that implement strategies to increase CRF and/or recovery time during working hours are needed in order to investigate the effectiveness and efficiency of such strategies in reducing cardiovascular risk among workers exposed to high OPA.

## Figures and Tables

**Table 1:** Descriptive characteristics of the study population (N = 309).

Demographic Characteristics	N (%)	Mean (SD)
Age (years)		38.5 (11.2)
Sex Female Male	178 (57.6) 131 (42.4)	
Educational level Low (until primary school) Medium (secondary school and/or 1 to 2 years of specialization) High (university or university college)	49 (15.9) 102 (33) 158 (51.1)	
Marital status Married/living together with children Married/living together without children Single with children Single without children	135 (43.7) 78 (25.2) 24 (7.8) 53 (17.2)	
BMI (kg/m²)		24.7 (3.9)
Current smoker	68 (22.0)	
Alcohol consumption <7 units per week >7 units per week	248 (80.3) 61 (19.7)	
Use of medication for heart condition and/or high blood pressure	16 (5.2)	
Job type Manufacturing sector Service sector Skilled worker Factory sector Unskilled worker	153 (49.5) 4 (1.3) 23 (7.4) 105 (34) 24 (7.8)	
Work schedule Shift Day job	198 (64.1) 109 (35.3)	
Workhours per week		36.9 (5.9)
Self-reported physical work demands		2.4 (0.7)
**Accelerometer-assessed information**	**N (%)**	**Mean (SD)**
Valid accelerometer wear-days		2.8 (0.9)
Work time (min/day)		467 (65)
Leisure time (min/day)		458 (98)
Sleep time (min/day)		447 (68)
**Accelerometer-assessed behaviours (absolute numbers)**	**N (%)**	**Mean (SD)**
SB work (min/day)		150 (103)
Standing work (min/day)		171 (75)
LIPA work (min/day)		78 (47)
MVPA work (min/day)		67 (37)
SB leisure (min/day)		272 (88)
Standing leisure (min/day)		97 (56)
LIPA leisure (min/day)		43 (20)
MVPA leisure (min/day)		44 (25)

Abbreviations: BMI = body mass index; SD = standard deviation; *n* = number of participants; SB = sedentary behaviour; LIPA = low-intensity physical activity; MVPA = moderate-to-vigorous physical activity.

**Table 2:** Compositional means for occupational and leisure time behaviours.

Occupational Behaviours	Minutes	% of Total Work Time
Sedentary behaviour	134.7	29.3
Standing	181.6	39.5
Low-intensity PA	76.1	16.5
Moderate-to-vigorous PA	67.6	14.7
**Leisure Time Behaviours**	**Minutes**	**% of Total Leisure Time**
Sedentary behaviour	282.8	61.5
Standing	93.9	20.4
Low-intensity PA	42.3	9.2
Moderate-to-vigorous PA	41.0	8.9

Abbreviation: PA = physical activity.

**Table 3:** Compositional multiple linear regression analyses of the relation between ilr₁ (first ilr) coordinates and cardiorespiratory fitness (CRF) (compositional models).

Compositional Regression Models (Work Time)	β	SE	t-Value	*p*
Model 1 (Ilr₁ = SB: geometric mean of remaining behaviours)	4.66	1.65	2.82	**<0.01**
Model 2 (Ilr₁ = Standing: geometric mean of remaining behaviours)	5.45	3.03	1.80	0.07
Model 3 (Ilr₁ = LIPA: geometric mean of remaining behaviours)	−4.74	3.97	−1.20	0.23
Model 4 (Ilr₁ = MVPA: geometric mean of remaining behaviours)	−5.37	3.41	−1.57	0.12
**Compositional Regression Models (Leisure Time)**	**β**	**SE**	**t-Value**	***p***
Model 1 (Ilr₁ = SB: geometric mean of remaining behaviours)	−11.84	2.40	−4.94	**<0.001**
Model 2 (Ilr₁ = Standing: geometric mean of remaining behaviours)	−1.18	4.02	−0.29	0.77
Model 3 (Ilr₁ = LIPA: geometric mean of remaining behaviours)	0.03	5.31	0.01	0.99
Model 4 (Ilr₁ = MVPA: geometric mean of remaining behaviours)	12.99	3.21	4.05	**<0.001**

Abbreviations: SB = sedentary behaviour; LIPA = low-intensity physical activity; MVPA = moderate-to-vigorous physical activity; Ilr₁ = first ilr-coordinate; SE = Standard Error; *p* = *p*-value. Models adjusted for age, sex, educational level, smoking, BMI, MVPA expressed as ilrs, and physical work demands. Significant at *p* < 0.05 and indicated in **bold**.

## Supplementary Materials

The following are available online at https://www.mdpi.com/1660-4601/17/21/7929/s1: Table S1: Estimated differences in CRF levels (95% confidence intervals) associated with one-to-remaining reallocations of different behaviours during work and leisure time separately among 309 workers involved in physically demanding jobs. Table S2: Estimated differences in CRF levels (95% confidence intervals) associated with one-to-one reallocations of different behaviours during work and leisure time separately among 309 workers involved in physically demanding jobs.
