# Supplementary material for: The Relation between Domain-Specific Physical Behaviour and Cardiorespiratory Fitness: A Cross-Sectional Compositional Data Analysis on the Physical Activity Health Paradox Using Accelerometer-Assessed Data
Source: Int J Environ Res Public Health. 2020 Oct 29;17(21):7929. doi: 10.3390/ijerph17217929 (PMC7662405; doi:10.3390/ijerph17217929)
Supplement: Supplementary file 1 [file ijerph-17-07929-s001.pdf]

Table S1. Estimated difference in CRF levels (95% confidence intervals) associated with one-to-remaining reallocations of different behaviours during work and leisure time separately among 309 workers involved in physically demanding jobs.

Difference in work behaviour (min/day)	Estimated difference in CRF (Physical fitness index, Harvard Step test) [95% CI]			
	**SB to remaining**	**Standing to remaining**	**LIPA to remaining**	**MVPA to remaining**
−30 min	**−1.37 [−2.33; −0.42]**	−1.34 [−2.80; 0.13]	2.37 [−1.53; 6.27]	3.07 [−0.77; 6.90]
−20 min	**−0.89 [−1.51; −0.27]**	−0.88 [−1.84; 0.08]	1.46 [−0.95; 3.87]	1.86 [−0.47; 4.19]
−10 min	**−0.43 [−0.74; −0.13]**	−0.43 [−0.91; 0.04]	0.68 [−0.44; 1.81]	0.86 [−0.22; 1.94]
0				
+10 min	**0.42 [0.13;0.70]**	0.43 [−0.04; 0.89]	−0.62 [−1.63; 0.40]	−0.76 [−1.71; 0.19]
+20 min	**0.82 [0.25;1.38]**	0.85 [−0.08; 1.77]	−1.18 [−3.12; 0.76]	−1.45 [−3.26; 0.36]
+30 min	**1.20 [0.36;2.04]**	1.26 [−0.12; 2.64]	−1.70 [−4.50; 1.10]	−2.08 [−4.67; 0.52]
**Leisure time (min/day)**				
−30 min	**2.75 [1.66;3.85]**	0.48 [−2.70; 3.65]	−0.03 [−11.83; 11.77]	**−15.57 [−23.12; −8.01]**
−20 min	**1.85 [1.11;2.59]**	0.30 [−1.70; 2.30]	−0.02[−6.23; 6.20]	**−8.05 [−11.95; −4.14]**
−10 min	**0.93 [0.56;1.30]**	0.14 [−0.81; 1.10]	−0.01 [−2.66; 2.65]	**−3.41 [−5.06; −1.75]**
0				
+10 min	**−0.95 [−1.33; −0.57]**	−0.13 [−1.01; 0.75]	0.01 [−2.13; 2.15]	**2.73 [1.40; 4.05]**
+20 min	**−1.93 [−2.70; −1.16]**	−0.26 [−1.96; 1.45]	0.01 −3.94; 3.96]	**5.02 [2.58; 7.46]**
+30 min	**−2.94 [−4.11; −1.77]**	−0.37 [−2.86; 2.11]	0.01 [−5.51; 5.54]	**7.01 [3.61; 10.42]**

Abbreviations: CRF = cardiorespiratory fitness; SB = sedentary behaviour; LIPA = low intensity physical activity; MVPA= moderate-to-vigorous physical activity. Statistically significance, when 95% CI did not include zero, is marked in **bold**.

Table S2. Estimated difference in CRF levels (95% confidence intervals) associated with one-to-one reallocations of different behaviours during work and leisure time separately among 309 workers involved in physically demanding jobs.

**Reallocation work**	Estimated difference in CRF (Physical fitness index, Harvard Step test)		
	10 min	20 min	30 min
SB to standing	−0.30 [−1.54; 0.95]	−0.16 [−0.98; 0.67]	−0.06 [−0.47; 0.35]
SB to LIPA	**−0.82 [−1.63; −0.01]**	**−1.61 [−3.14; −0.07]**	**−2.38 [−4.57; −0.19]**
SB to MVPA	**−0.95 [−1.87; −0.04]**	**−1.85 [−3.60; −0.11]**	**−2.72 [−5.24; −0.21]**
Standing to SB	0.02 [−0.39; 0.44]	0.01 [−0.83; 0.84]	−0.04 [−1.30; 1.22]
Standing to LIPA	−0.77 [−1.82; 0.27]	−1.51 [−3.53; 0.51]	−2.22 [−5.15; 0.72]
Standing to MVPA	**−0.91 [−1.75; −0.07]**	**−1.75 [−3.36; −0.15]**	**−2.56 [−4.86; −0.26]**
LIPA to SB	0.87 [−0.06; 1.79]	1.81 [−0.19; 3.82]	2.87 [−0.43; 6.17]
LIPA to standing	0.83 [−0.32; 1.98]	1.75 [0.70; 4.19]	2.78 [−1.16; 6.72]
LIPA to MVPA	−0.06 [−1.69; 1.56]	0.05 [−3.26; 3.36]	0.35[−4.78; 5.49]
MVPA to SB	**1.03 [0.00; 2.06]**	2.19 [−0.04; 4.43]	3.54 [−0.15; 7.22]
MVPA to standing	**0.99 [0.04; 1.96]**	**2.12 [0.03; 4.21]**	3.45 [−0.03; 6.91]
MVPA to LIPA	0.24 [−1.40; 1.87]	0.67 [−2.68; 4.02]	1.36 [−3.89; 6.61]
**Reallocation leisure**			
SB to standing	0.27 [−0.48; 1.01]	0.55 [−0.88; 1.99]	0.87 [−1.21; 2.94]
SB to LIPA	0.37 [−1.57; 2.32]	0.76 [−2.79; 4.31]	1.16 [−3.76; 6.09]
SB to MVPA	**2.82 [1.59; 4.06]**	**5.22 [2.97; 7.47]**	**7.33 [4.20; 10.45]**
Standing to SB	−0.24 [−1.06; 0.58]	−0.46 [−2.19; 1.28]	−0.64 [−3.42; 2.14]
Standing to LIPA	0.12 [−2.42; 2.67]	0.25 [−4.59; 5.10]	0.41 [−6.62; 7.44]
Standing to MVPA	**2.57 [1.27; 3.87]**	**4.71 [2.24; 7.18]**	**6.57 [2.96; 10.18]**
LIPA to SB	−0.36 [−2.82; 2.10]	−0.72 [−6.55; 5.12]	−1.06 [−12.29; 10.17]
LIPA to standing	−0.11 [−3.10; 2.88]	−0.21 [−7.04; 6.62]	−0.31 [−12.96; 12.33]
LIPA to MVPA	2.45 [−0.89; 5.78]	4.45 [−2.93; 11.83]	6.15 [−7.16; 19.45]
MVPA to SB	**−3.50 [−5.06; −1.94]**	**−8.22 [−11.94; −4.50]**	**−15.82 [−23.10; −8.54]**
MVPA to standing	**−3.25 [−4.80; −1.69]**	**−7.72 [−11.37; −4.07]**	**−15.07 [−22.16; −7.99]**
MVPA to LIPA	**−3.14 [−6.27; −0.01]**	**−7.51 [−14.01; −1.02]**	**−14.77 [−25.73; −3.82]**

Abbreviations: CRF = cardiorespiratory fitness; SB = sedentary behaviour; LIPA = low intensity physical activity; MVPA = moderate-to-vigorous physical activity. Statistically significance, when 95% CI did not include zero, is marked in **bold**.
